# Vascular Permeability and Drug Delivery in Cancers

**DOI:** 10.3389/fonc.2013.00211

**Published:** 2013-08-15

**Authors:** Sandy Azzi, Jagoda K. Hebda, Julie Gavard

**Affiliations:** ^1^CNRS, UMR8104, Paris, France; ^2^INSERM, U1016, Paris, France; ^3^Sorbonne Paris Cite, Universite Paris Descartes, Paris, France

**Keywords:** VEGF, permeability, VE-cadherin, endothelial barrier, tumor angiogenesis

## Abstract

The endothelial barrier strictly maintains vascular and tissue homeostasis, and therefore modulates many physiological processes such as angiogenesis, immune responses, and dynamic exchanges throughout organs. Consequently, alteration of this finely tuned function may have devastating consequences for the organism. This is particularly obvious in cancers, where a disorganized and leaky blood vessel network irrigates solid tumors. In this context, vascular permeability drives tumor-induced angiogenesis, blood flow disturbances, inflammatory cell infiltration, and tumor cell extravasation. This can directly restrain the efficacy of conventional therapies by limiting intravenous drug delivery. Indeed, for more effective anti-angiogenic therapies, it is now accepted that not only should excessive angiogenesis be alleviated, but also that the tumor vasculature needs to be normalized. Recovery of normal state vasculature requires diminishing hyperpermeability, increasing pericyte coverage, and restoring the basement membrane, to subsequently reduce hypoxia, and interstitial fluid pressure. In this review, we will introduce how vascular permeability accompanies tumor progression and, as a collateral damage, impacts on efficient drug delivery. The molecular mechanisms involved in tumor-driven vascular permeability will next be detailed, with a particular focus on the main factors produced by tumor cells, especially the emblematic vascular endothelial growth factor. Finally, new perspectives in cancer therapy will be presented, centered on the use of anti-permeability factors and normalization agents.

## Vascular Permeability in Cancers

### Vascular barrier organization

Endothelial cells, pericytes, smooth muscle cells, and the basal membrane collectively form the blood vascular wall, which ensures selective exchanges between plasma and irrigated tissues. The passage of macromolecules, fluids, and cells through this endothelial barrier can occur either through (transcellular) or between cells (paracellular) (1). The ability to pass from the interstitial space to the blood compartment, and *vice versa* depends on charge, size, and binding characteristics.

**Figure 1:**
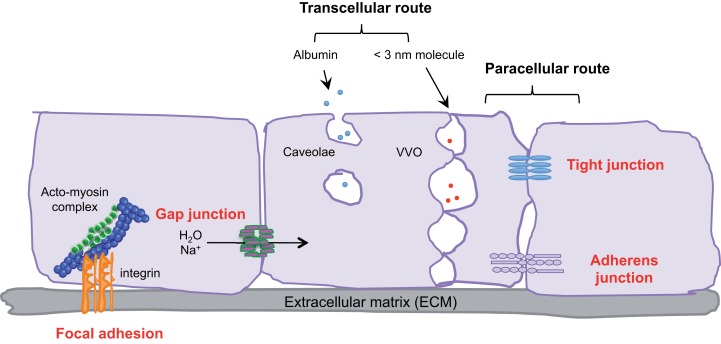
**Transcellular and paracellular pathways in endothelial cells**. The passage of cells and macromolecules through the endothelial barrier can occur through transcellular (vesicular vacuolar organelles) or paracellular (tight and adherens junctions) pathways. Gap junctions ensure water and ion transport. Moreover, endothelial cells are anchored and connected to the extracellular matrix (ECM) through integrin-based adhesion complexes, namely focal adhesions.

Small molecules (inferior to 3 nm) are commonly transported by the transcellular route, which requires a system of trafficking vesicles, called vesicular vacuolar organelles (VVOs) (Figure 1). Several permeability factors, such as vascular endothelial growth factor (VEGF) and histamine have been demonstrated to activate VVOs and to orchestrate vascular homeostasis (2). These VVOs comprise, among other things, clustered caveolae, and rely on caveolin-1 protein function, that notably guarantees albumin transport. Interestingly, caveolin-1 plays a dual regulatory role in microvascular permeability by stabilizing caveolae structures and allowing caveolar transcytosis, while acting as a negative regulator through endothelial nitric oxide synthase (eNOS) (3, 4).

**Figure 2:**
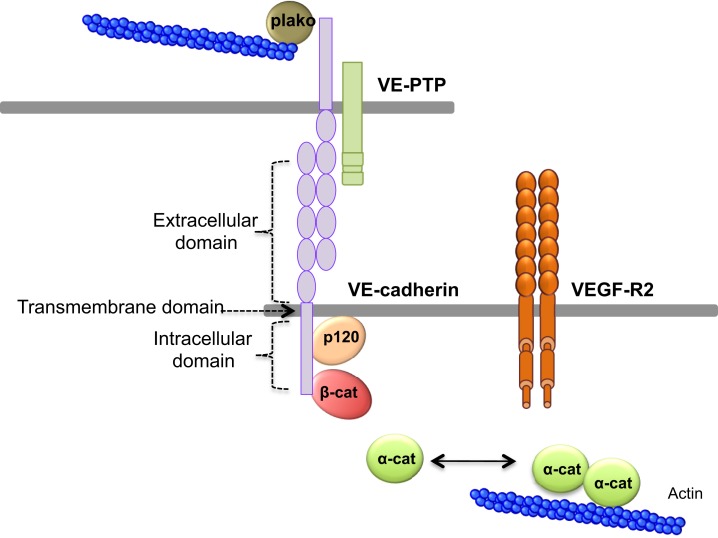
**VE-cadherin adhesive complex**. VE-cadherin mediates the adhesion between endothelial cells in calcium-dependent manner. VE-cadherin is constituted of an extracellular domain, which allows homophilic interaction in *trans*. The transmembrane domain participates to lateral clustering in *cis*. The intracellular domain of VE-cadherin binds p120-catenin (p120), and β-catenin (β-cat), which participates to VE-cadherin membrane retention. Actin cytoskeleton is anchored to VE-cadherin via α-catenin (α-cat) or plakoglobin (plako). In addition, VE-cadherin can bind VEGF-R2 (vascular endothelial growth factor receptor 2) and VE-PTP (vascular endothelial phosphotyrosine phosphatase).

Cells and macromolecules larger than 3 nm use the paracellular pathway, which is mediated by the coordinated opening and closing of endothelial cell–cell junctions. Adherens (AJ) and tight (TJ) junctions maintain the restrictiveness of the barrier, while gap junctions connect adjacent endothelial cells. Gap junctions are responsible for water and ion transport but do not contribute significantly or directly to the establishment of vascular barriers (Figure 1). Among AJ proteins, the most important is vascular endothelial cadherin (VE-cadherin), which is exclusively expressed in vessels (1, 5). In mice, VE-cadherin gene deletion results in early embryonic lethality due to massive vascular defects, while loss of its function provokes a hyperpermeability phenotype in adults (6, 7). VE-cadherin comprises five immunoglobulin-like domain repeats in its extracellular region, one single-pass transmembrane domain and a short cytoplasmic tail. While the extracellular domain confers Ca^++^ dependency and allows homophilic interaction in *trans* (i.e., between cadherins on neighboring cells), the transmembrane domain participates in lateral clustering within the same cell (*cis*) (8). The cytoplasmic part of VE-cadherin binds to proteins from the armadillo-repeat gene family, namely p120-catenin and either β-catenin or plakoglobin (γ-catenin). This complex serves to strengthen adhesion forces and allows dynamic contacts (Figure 2). p120-catenin interacts with the juxtamembrane part of the VE-cadherin cytoplasmic domain and is involved in its retention at the cell surface, while β/γ-catenins, on the other hand, act as constitutive partners of VE-cadherin, bound to its carboxy-terminal part (9, 10). Importantly, VE-cadherin is also connected to the actin cytoskeleton through the actin-binding protein, α-catenin (5). Other adhesive proteins that accumulate in or close to AJ, include N-cadherin, platelet-endothelial cell adhesion molecule (PECAM-1), and junctional adhesion molecules (JAMs).

Tight junctions participate in endothelial cell cohesion and block molecule diffusion along the apical and basolateral poles (11). They rely on transmembrane adhesion proteins (occludin and claudins), JAM family proteins, and intracellular connectors, including ZO-1, -2, -3 (Zonula Occludens). First, occludin and claudins contain four transmembrane domains with N- and C-terminal intracellular parts. Second, JAM-A belongs to the immunoglobulin superfamily with one intracellular short domain, one single transmembrane domain, and two extracellular immunoglobulin-like domains. Third, the ZO proteins contain three PDZ (post synaptic density protein PSD95, Drosophila disk large tumor suppressor Dlg1, and zonula occludens-1 protein zo-1), one SH3 (SRC homology 3) and one guanylyl kinase-like domains (11). Contrary to VE-cadherin, deletion of the claudin-5 gene does not impair mouse embryo development, but rather leads to post-natal death caused by a defective blood-brain barrier (12). Thus, VE-cadherin is instrumental in vascular barrier integrity, while claudins may have a more restrictive role (13). Nevertheless AJ and TJ are functionally and structurally linked and can influence each other (14, 15).

Within blood vessels, endothelial cells are interactively anchored to the extracellular matrix (ECM) through integrin-based adhesion complexes, namely focal adhesions (Figure 1). Indeed, integrins bridge the ECM to the acto-myosin contractility apparatus (16), and allow endothelial cells to adapt to extracellular signals and cues (e.g., shear stress and secreted molecules). From a molecular standpoint, Rho-GTPase activation, stress fiber formation, and acto-myosin contraction are finely tuned through integrin adhesion and collectively contribute to the modulation of endothelial junction integrity (17, 18). More recently, it was demonstrated that the integrin-associated focal adhesion tyrosine kinase (FAK) contributes to the impairment of vascular barrier function (19). Indeed, VEGF-induced FAK activation was shown to lead to VE-cadherin/FAK interaction in association with β-catenin phosphorylation on tyrosine Y142, resulting in VE-cadherin/β-catenin dissociation, junction opening, and endothelial barrier disruption.

Hence, vascular barrier properties depend on both structural (basal membrane, smooth muscle cells, endothelial cells) and functional (VVO, AJ, TJ) features. To endorse this role, endothelial cell adhesion has to be tightly regulated. Indeed, aberrant and uncontrolled increase of vascular permeability can participate in the progression of many pathological states, such as chronic inflammatory diseases, diabetes, and tumor angiogenesis.

### Vascular leakage in the tumor microenvironment

Compared to normal tissues, tumor vasculature is immature and exhibits structural abnormalities, such as dilatation, saccular formation, and a hyper-branched and twisted pattern. Moreover, solid tumors usually present few to none functional lymphatic vessels (20, 21). Many molecular and cellular factors contribute to this morphological and functional failure, in which vascular permeability is central. Rapidly growing tumors secrete an abundance of different factors (VEGF, chemokines, and others) that govern uncontrolled angiogenesis. In such microenvironments, most of the criteria that define the endothelial barrier properties are circumvented.

First, tumor vessels are characterized by extensive angiogenesis, i.e., neovessel formation from pre-existing vascular networks. In this scenario, tumor endothelial cells have a proliferation rate 50–200 times faster than that of normal quiescent endothelial cells (22). They also have to migrate and rearrange into vascular tubules, dedicated to fuel the tumor mass. This high endothelial plasticity in the constantly remodeled vascular wall is accompanied by elevated permeability. Tumor vessel hyperpermeability correlates with faint VE-cadherin expression, opening of paracellular junctions, and transcellular holes formation (23). In the course of tumor growth, the direct consequence of hyperpermeable vessels is plasma membrane protein extravasation and formation of a provisory matrix to allow endothelial cell sprouting and formation of new vessels (24).

**Figure 3:**
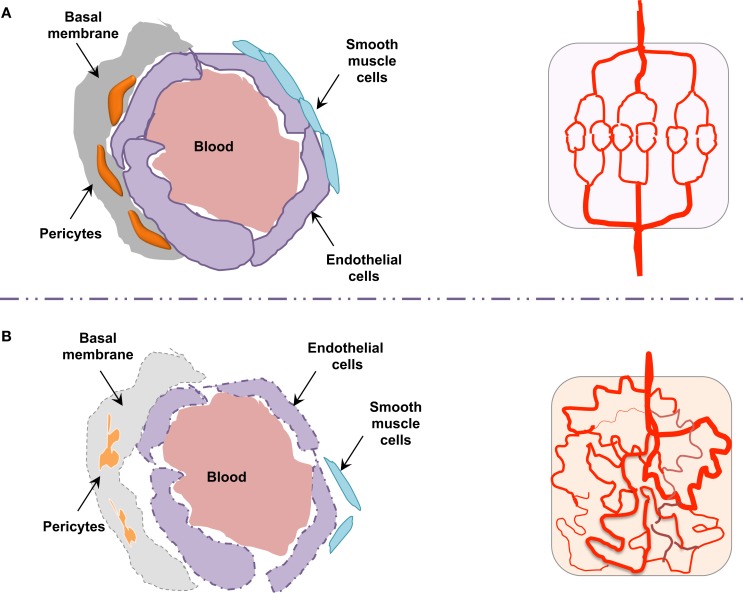
**Endothelial barrier in normal and tumor vessels**. The endothelial barrier structure differs in normal **(A)** and tumor **(B)** blood vessels. Contrary to normal vessels, the tumor vasculature pattern is extremely disorganized and anarchic, presents morphological and structural difference, i.e., weak association between endothelial cells, abnormal shapes of pericytes, lack of smooth muscles, as well as basal membrane modification.

Morphologically, the pericytes surrounding tumor vessels are abnormally shaped and are weakly associated with endothelial cells (25). In addition, tumor blood vessels lack smooth muscles (Figure 3). Similarly, the basal membrane can be either unusually thick or totally absent (26). In these conditions, resistance to blood flow is increased, and thereby the efficacy in tumor blood supply is reduced. As a consequence, despite a high microvessel density, tumors are poorly vascularized with hyperpermeable vasculature. This could lead *in fine* to the accumulation of metabolic products (lactic and carbonic acids) and extracellular pH decrease (27). Tumor vessel defects also quell oxygen supply, frequently causing hypoxia in the tumor microenvironment. Hypoxia, in turn, supports tumor angiogenesis through the hypoxia-inducible transcription factors (HIF), and further elevates the expression of pro-angiogenic molecules, such as VEGF, TNF (tumor necrosis factor), and PDGF (platelet-derived growth factor). Interestingly, because of its involvement in chemo- and radio-resistance, as well as metastasis, hypoxia has been suggested as an adverse prognostic factor (28).

Within the tumor microenvironment, the ECM undergoes significant compositional modifications most notably by increasing the levels of expression of collagen-1, matrix metalloproteases (MMP)-1 and -2, and laminin-5 (29). For instance, collagen-1 deposit increases ECM stiffness, and this is related with poor prognosis and higher metastasis potential (30). In addition, ECM stiffness enhances integrin expression and promotes focal adhesion signaling, and consequently influences tumor cell malignancy (31).

In summary, abnormal blood vessels and lack of lymphatic vessels in tumors, as well as increased ECM stiffness and relatively high interstitial fluid pressure (IFP) collectively contribute to the functional outcome called enhanced permeability and retention (EPR). This phenomenon facilitates both macromolecule extravasation and retention. Whereas normal vessels form a selective barrier, limiting cell and macromolecule passage, the tumor vasculature is extremely leaky and not restrictive. Consequently, although these features could benefit to tumor angiogenesis and growth, anti-tumor drug delivery is rather limited.

### Impact on drug delivery

To gain in bioavailability and selectivity toward tumor cells, therapeutic molecules must counteract biological and physical barriers, among which are endothelial transport and blood flow. Drug efflux pumps are one of the main obstacles that anti-cancer drugs must overcome. These transporters are highly expressed in a large panel of cancer cells, as well as in the blood-brain barrier, where they ensure drug detoxification (32, 33).

However, tumor vessels cannot ensure correct tumor blood perfusion, since they are structurally aberrant and hyperpermeable (24). The pressure difference between vessels and surrounding tissues constitutes also an important physical barrier. Upon vascular leakage, transcapillary interstitial fluid flow decreases and IFP increases resulting in poor drug penetration through tumor vessels (21). In addition to blood vessel leakage, both the absence of a functional lymphatic system and increased ECM-frictional resistance also lead to tumor IFP increase (34). This ultimately provokes disruption in blood flow directions, again limiting drug delivery.

Importantly, tumor drug delivery can be tailored by changing the size and charge of the delivered molecule. Of interest, molecules larger than 40 kDa cannot be passively eliminated through renal clearance and are unable to cross normal blood vessels through endothelial junctions; however, they could easily penetrate tumors through leaky vessels. EPR of tumor vessels permits the passage of molecules ranging from 40 to 70 kDa, thus, in association with other properties such as the ability to traverse relatively long distances, prolonged plasma half-life and slow clearance, these larger molecule have been proposed to be the most appropriate for specific tumor delivery (35, 36). In addition, due to the negative charges of the vessel luminal face, the use of cationic therapeutic molecules may also favor vascular accumulation, which in turn can elevate tumor drug concentration (37).

Thus, although drug delivery is strongly impaired in tumors because of structural and functional vascular defects, some of these constraints, such as vessel leakiness, can be exploited for curative purposes.

## Molecular Mechanisms Involved in Vascular Permeability

As presented above, endothelial barrier integrity ensures vascular and tissue homeostasis. In cancers, deregulation of this fine-tuned function leads to the formation of a chaotic blood vessel network associated with elevated permeability. We will now detail the molecular mechanisms involved in tumor-driven vascular permeability, focusing on the main factors produced by tumor cells, such as VEGF and chemokines. This knowledge could open new avenues for drug design.

### Vascular endothelial growth factor

Vascular endothelial growth factor belongs to the family of platelet-derived growth factors and was originally referred to as vascular permeability factor (38). It is a homodimeric glycoprotein of which several forms have been described in mammals, these are: VEGF-A, B, C, D, and the placenta growth factor PlGF. Among these, VEGF-A is the most commonly studied and better described in literature. Various cell types, such as endothelial cells, smooth muscle cells, fibroblasts, and immune cells (macrophages, lymphocytes, neutrophils, and eosinophils) can produce and release VEGF within the environment. In turn, VEGF can act in both an autocrine and paracrine manner. In cancers, tumor cells constitute an important source of VEGF. VEGF stimulates endothelial cell growth and promotes vasculogenesis and angiogenesis. It also increases vascular permeability, its first described function, in many tissues, and plays a crucial role in tumor vasculature development (22). VEGF intracellular signaling is mediated by three tyrosine kinase-receptors, namely VEGF-R1, -R2, and -R3, as well as co-receptors such as neuropilins. The binding of the ligand to its cognate receptors induces their dimerization, autophosphorylation, and subsequent signal transduction (39). VEGF-A interacts with both VEGF-R1 and -R2, but only VEGF-R2 is directly involved in normal and pathological vascular permeability (40). However, VEGF-R1 is reported to act as a regulator of VEGF-R2 signaling, and thus might indirectly regulate vascular permeability.

**Figure 4:**
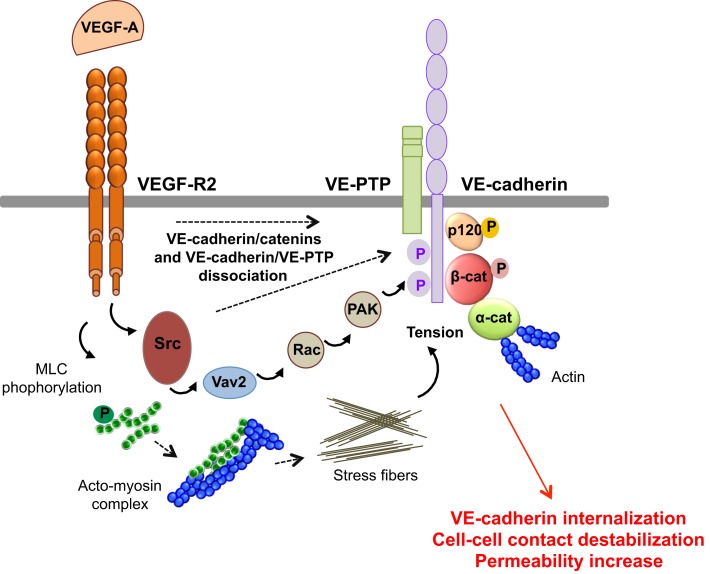
**Molecular pathways involved in VEGF-endothelial permeability**. VEGF-A stimulation induces VEGF-R2 dimerization and the sequential activation of Vav2, Rac, and PAK, through Src. This results in the serine phosphorylation of VE-cadherin by PAK, and its subsequent internalization into clathrin-coated pits. VEGF can also trigger the tyrosine phosphorylation of VE-cadherin and of its binding partners β-catenin (β-cat) and p120, in a Src-dependent fashion. In addition, VEGF-A decreases the VE-cadherin/p120-catenin association and promotes VE-cadherin endocytosis. VEGF-A also induces the phosphorylation of myosin light chains (MLC), which produces stress fibers that exert tension on intercellular junctions, thus weakening cell–cell contacts. Finally, VEGF-A stimulation causes the dissociation of VE-PTP/VE-cadherin and triggers loss of adhesion and permeability increase.

VEGF-A promotes vascular permeability by disruption of AJ and TJ, resulting in transient opening of endothelial cell–cell contacts (5, 14) (Figure 4). Indeed, VEGF-A promotes tyrosine phosphorylation of VE-cadherin and of its binding partners β-catenin, plakoglobin, and p120, in a Src-dependent mechanism (41). Consistent with this, VE-cadherin phosphorylation is inhibited in Src-deficient mice (41). VE-cadherin can also associate with VEGF-R2 and inhibit its phosphorylation and subsequent internalization (42). This association potentiates the phosphorylation of AJ components by Src, thus impairing endothelial barrier integrity and favoring tumor cell extravasation and dissemination in pathological models (43). The VE-cadherin/VEGF-R2 association also contributes to VE-cadherin-induced contact inhibition of cell growth and requires the β-, but not p120-catenin, binding domain of VE-cadherin (42, 44). In addition, VEGF-A mediates VE-cadherin phosphorylation and internalization via the sequential activation of Src, the guanine nucleotide exchange factor Vav2, the Rho-GTPase Rac, and its downstream effector, the serine-threonine kinase PAK (Figure 4). This signaling pathway culminates in the PAK-dependent phosphorylation of VE-cadherin, which directs its internalization (45). Moreover, VEGF signaling decreases VE-cadherin/p120-catenin association promoting clathrin-dependent VE-cadherin endocytosis (46). Indeed, p120 binding to VE-cadherin prevents its internalization, while its silencing by siRNA leads to VE-cadherin degradation, and loss of cell–cell contacts (10, 47). The expression of VE-cadherin mutants that compete with the endogenous molecule for binding with p120, triggers VE-cadherin degradation, suggesting that p120 might act as plasma membrane retention signal. More recently, a motif within VE-cadherin was identified to be responsible for VE-cadherin/p120 coupling and endocytosis sorting (48). In this context, VE-cadherin-mediated cell–cell contacts are stabilized by the small GTPase Rap1 and its effector, the cyclic adenosine monophosphate (cAMP)-activated guanidine exchange factor Epac (49, 50). Small GTPases also regulate myosin light chain (MLC) phosphorylation, acto-myosin contractility, and endothelial permeability (51). Indeed, VEGF induces the phosphorylation of MLC that results in the formation of stress fibers which exert centripetal tension on intercellular junctions (52).

Of note, endothelial permeability can also be regulated through changes in the expression of AJ and TJ components (15, 53). For example, VEGF signaling through VEGF-R2 induces the expression of SRF (serum response factor), which is important for VE-cadherin expression (54). Indeed, SRF knockdown in mice reduces VE-cadherin expression and angiogenesis. Furthermore, claudin-5 expression is regulated by VE-cadherin, confirming that the latter is instrumental in controlling endothelial barrier function (15). Recently, it has been described that VEGF is involved in claudin-5 down-regulation in peritoneal endothelium, inducing ascites in ovarian cancer patients (55).

### Interleukin-8

Cytokines are key drivers of immune responses and play important roles in cancer progression. Among these, the chemokine IL-8 (CXCL8, CXC chemokine ligand 8) is overexpressed and secreted by cancerous cells. Of note, their cognate G-protein-coupled receptors (GPCR), CXCR1 and CXCR2, are expressed on endothelial cells, tumor cells, and neutrophils/tumor-associated-macrophages, indicative of pleiotropic activities of IL-8. Activation of IL-8 endothelial receptors is known to promote angiogenic responses, through enhanced proliferation, survival, and migration (56). Furthermore, intratumoral IL-8 concentration is proposed to chiefly cause neutrophil recruitment into the tumor microenvironment and to promote metastasis (57). Besides its chemotactic role, IL-8 arose as an essential factor of angiogenesis and increased vascular permeability (58). Indeed, IL-8 can provoke VEGF-R2 phosphorylation and transactivation, which in turn result in both Src and RhoA activation, leading to endothelial gap formation, and elevated permeability (59). IL-8 can also increase permeability in mouse and human endothelial cells via a VEGF-R2 independent mechanism (60). IL-8 initiates a signaling route through CXCR2/Rac/PI3Kγ that triggers the phosphorylation and subsequent internalization of VE-cadherin, thereby promoting increased permeability. Moreover, blockade of CXCR2 and PI3Kγ with pharmacological inhibitors or by RNA interference (RNAi), limits IL-8-induced neovascularization and vessel leakage (60). In glioblastoma, cancer cells were found to secrete high concentrations of IL-8, which was further demonstrated to function as a key factor involved in tumor-induced permeability *in vitro*, and to signal to brain microvascular endothelial cells via CXCR2, promoting VE-cadherin cell–cell junction remodeling, and elevated permeability (61). Similarly, in prostate cancers, IL-8 secretion is associated with increased Akt expression and activation, which impacts on endothelial cell survival, angiogenesis, and cell migration (62).

### Transforming growth factor-β1

TGF-β1 is a multifunctional polypeptide member of the transforming growth factor beta superfamily. It regulates the production of cytokines and ECM components, and is involved in diverse biological processes, such as proliferation and differentiation in many cell types (63–65). Within the tumor microenvironment, macrophages, mesenchymal, and cancer cells secrete TGF-β1 under hypoxic and inflammatory conditions. TGF-β1 was suggested to act as a potent inducer of angiogenesis, since its increased expression correlates with high microvessel density and poor prognosis in various types of cancers (66). TGF-β1 also augments vascular permeability by altering cell–cell contacts. This is thought to involve p38 mitogen-activated protein kinase (MAPK) and RhoA signaling cascades, which in turn modulate ECM adhesion and lead to the loss of endothelial-barrier integrity and function (67). In primary breast tumors, TGF-β1 activity is associated with an increased risk of lung metastasis. Indeed, angiopoietin-related protein 4 (ANGPTL4), a TGF-β1 target gene, disrupts endothelial cell–cell junctions, and facilitates the extravasation of breast cancer cells (68). Moreover, TGF-β1 induces the expression of VEGF in fibroblasts (69), whereas it inhibits angiopoietin-1, an anti-permeability factor, therefore exacerbating tumor-associated vascular leakage (70). In addition, TGF-β1 potentiates the secretion and activation of MMPs (71).

### Stromal cell-derived factor 1

Stromal cell-derived factor 1, also known as CXCL12, is a member of the α-chemokine subfamily and the ligand for the GPCR CXCR4. In adulthood, SDF-1 was implicated in angiogenesis by recruiting endothelial progenitor cells from the bone marrow (72). SDF-1 is highly expressed in a number of cancers and is associated with tumor extravasation and increased metastases (73, 74). CXCR4 expression also corroborates with metastatic properties of breast cancer cells (75). Indeed, CXCR4 levels were found to be higher in malignant breast tumors in comparison to those of normal healthy counterparts. *In vivo*, neutralizing CXCR4/SDF-1 signaling axis significantly impaired breast cancer cell extravasation and propagation (75, 76). SDF-1 can also mediate endothelial permeability via CXCR4, as for instance, SDF-1 stimulation of breast cancer cells *in vitro* increased their passage across the endothelial barrier. This effect is dependent on both PI3K/Akt and calcium signaling in endothelial cells (77). Inhibiting this pathway with anti-CXCR4 antibodies, on the other hand, decreased vascular leakage (77). Moreover, SDF-1 is involved in macrophage recruitment to breast tumors in mice, in response to chemotherapy (78). This action is believed to stimulate tumor blood vessel growth, counteracting the effects of the drug. Finally, it is to be noted that VEGF stimulates SDF-1 secretion and *vice versa* (79, 80). However, VEGF implication in SDF-1-induced permeability remains to be elucidated.

### Interleukin-10

IL-10, also known as human cytokine synthesis inhibitory factor, is an anti-inflammatory cytokine. The role of IL-10 in cancers, though well accepted, is vaguely understood (81). Indeed, IL-10 is suspected to exert both pro- and anti-tumor activities, and contradictory results have been reported regarding its involvement in tumor angiogenesis. On one hand, IL-10 could hamper angiogenesis and tumor growth in mice bearing VEGF-producing ovarian cancer (82), and suppress tumor growth and metastasis of human melanoma cells (83). On the other hand, other studies have suggested that IL-10 may promote angiogenesis in a melanoma cell model, by inhibiting macrophage functions and inducing tumor and vascular cell proliferation (84).

### Matrix metalloproteinases

Metalloproteinases are a large family of proteases that include MMP and ADAM (a disintegrin and metalloproteinase). MMPs belong to a family of zinc-containing endopeptidases that degrade various components of the ECM. Their aberrant over-expression correlates with cancer progression, cell invasion, and metastasis (85). Tumor-associated macrophages secrete VEGF and MMP-9, which are directly involved in both breast cancer and colorectal cancer cell invasion and metastasis (86). In addition, MMPs promote tumor progression by rearrangement of the ECM. Indeed, they trim cell adhesion molecules and degrade matrix proteins, favoring cell proliferation, and angiogenesis. MMP-7 can shed VE-cadherin, while MMP-2 and MMP-9 are involved in occludin proteolysis, thus enhancing endothelial permeability (87, 88). MMPs can also potentiate vascular leakage in a more indirect fashion, via cleavage and activation of chemokines such as IL-8, which is processed by MMP-9 (89, 90). Moreover, *in vitro* experiments have demonstrated a role for ADAM10 and ADAM17 in endothelial gap formation in response to cytokines. This is probably mediated by the cleavage of adhesion molecules within cell–cell junctions, including VE-cadherin and JAM-A (91).

### Semaphorins

Semaphorins correspond to a family of secreted and membrane-bound proteins that can act as both attractive and repulsive guidance molecules (92, 93). Besides their role in neural development, some of these molecules can modulate endothelial plasticity (94). Indeed, semaphorin 4D plays a positive role in endothelial migration and tumor angiogenesis (95, 96). In contrast, class 3 semaphorins, notably semaphorin 3A (S3A), and semaphorin 3E, are reported to operate as selective inhibitors of VEGF-induced angiogenesis (97–100). However, S3A and VEGF can also cooperate to induce vascular permeability (101). Indeed, S3A induces Akt phosphorylation through PI3K signaling, thus enhancing vascular permeability (101). In glioblastoma, the cancer stem-like cell sub-population expresses and secretes S3A *ex vivo* (102). In this context, S3A mediates endothelial cell–cell junction destabilization and elevates endothelial permeability (102). On a molecular level, S3A disrupts the VE-cadherin/PP2A complex, allowing VE-cadherin serine phosphorylation and subsequent internalization (45, 102). Consistent with this, inhibition of S3A by blocking antibody or by silencing RNAs has been demonstrated to abrogate these effects.

### Nitric oxide and peroxynitrite

Nitric oxide (NO) is a highly reactive free radical, which mediates a myriad of cellular reactions (103). NO is produced from l-arginine and oxygen by NO synthases (NOS). There are three major NOS isoforms: inducible NO synthase (NOS2/iNOS), eNOS (NOS3/eNOS), and neuronal NO synthase (NOS1/nNOS). NOS3 is constitutively expressed in endothelial cells, cardiac myocytes, and hippocampal pyramidal cells and is involved in suppressing platelet aggregation, maintaining vascular tone, inhibiting smooth muscle cell proliferation, and prompting angiogenesis (104). In cancers, NOS3 generates NO in blood vessels, which can favor endothelial proliferation, migration, and tumor progression (105, 106). Of note, NOS3 can be induced by VEGF in a MAPK/PLC-γ-dependent manner (107). NOS3 may also be involved in modulating vascular leakage. Indeed, it has been reported that eNOS translocation to the cytosol, but not to the Golgi, is associated with hyperpermeability *in vitro* and *in vivo* (108, 109). Stimulation of endothelial cells with platelet-activating factor (PAF) induces S-nitrosylation of β-catenin and p120 and significantly diminishes their association with VE-cadherin (110). Furthermore, VEGF treatment elicited S-nitrosylation of β-catenin at the Cys619 residue, within the VE-cadherin interaction site (111). Inhibition of β-catenin S-nitrosylation prevents NO-dependent dissociation of β-catenin from VE-cadherin and disassembly of AJ complexes, thereby inhibiting VEGF-mediated endothelial permeability (111). Moreover, oxidized products of NO, such as peroxynitrite (ONOO-), activate MMPs, which favor matrix rearrangement and endothelial permeability as discussed above. However, NO can induce cytotoxic effects on cancer cells. The balance between NO-mediated permeability and angiogenesis or apoptosis should thus be considered in tumor-targeted therapy (112).

In conclusion, endothelial permeability-mediated signaling pathways converge at the disruption and destabilization of cell–cell contacts, promoting AJ and TJ restructuration and subsequent opening of endothelial cell–cell junctions. We will now present the anti-permeability factors and normalization agents that may represent new perspectives in cancer therapy.

## Perspectives in Cancer Therapy

It is now well accepted that vascular permeability limits drug delivery thus restraining the efficacy of conventional therapies. New approaches aim now at diminishing both excessive angiogenesis and hyperpermeability. In this paragraph, perspectives in cancer therapy, such as the use of anti-permeability factors and blood vessel normalization agents will be discussed.

### Anti-permeability factors

**Figure 5:**
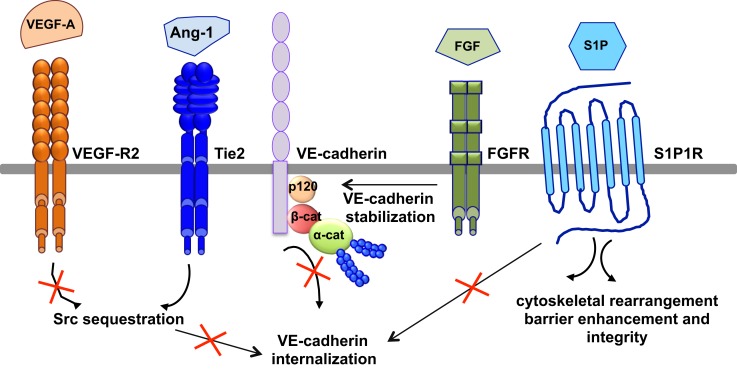
**Signaling pathways of anti-permeability factors**. The most relevant anti-permeability factors are angiopoietin-1 (Ang1), sphingosine-1-phosphate (S1P), and fibroblast growth factor (FGF). Ang1 activation of its cognate receptor, Tie2, elicits a signaling pathway and promotes Src sequestration; thus hindering VEGF-A signaling and VE-cadherin internalization. S1P signaling maintains vascular integrity by modulating VE-cadherin internalization, cytoskeletal rearrangement, barrier enhancement and integrity, through its cognate receptor S1P1R. FGF maintains the integrity of the VE-cadherin/p120-catenin complex, thus stabilizing VE-cadherin at the membrane.

The most relevant anti-permeability factors are angiopoietin-1 and its cognate receptor Tie2, sphingosine-1-phosphate (S1P), and fibroblast growth factor (FGF) (Figure 5).

Angiopoeitin-1 is a potent pro-angiogenic factor with the particularity of stabilizing blood vessels and counteracting VEGF-induced vascular permeability (63, 113). As mentioned above, VEGF elevates endothelial permeability via VE-cadherin adhesion destabilization in a Src-dependent mechanism (41, 45). Angiopoietin-1 elicits a signaling pathway through Tie2 that promotes the sequestration of Src though mammalian diaphanous (mDia) (114), thus hindering VEGF signaling and VE-cadherin internalization. In an intact endothelial monolayer, angiopoietin-1 promotes the interaction of Tie2 with the vascular endothelial protein tyrosine phosphatase VE-PTP (115, 116). VE-PTP associates with VE-cadherin and stabilizes it at the plasma membrane by blocking its tyrosine phosphorylation in response to VEGF-R2 activation (117). Angiopoeitin-1 also maintains the barrier integrity by increasing the association between VE-cadherin and plakoglobin (118), thus strengthening cell–cell contacts and limiting endothelial permeability. Consistently, VEGF signaling *in vivo* triggers the dissociation of VE-PTP from VE-cadherin, facilitating leukocyte extravasation and vessel leakage (119). Furthermore, angiopoeitin-1 balances VEGF pro-permeability actions by controlling NO release from endothelial cells. Indeed, it increases eNOS phosphorylation on Thr497, and subsequently reduces NO release and transendothelial permeability (120).

In addition to angiopoeitin-1, S1P, a biologically active phosphorylated lipid growth factor released from activated platelets, has emerged as an endothelial barrier protective agent. In both pulmonary artery and lung microvascular endothelial cells, S1P was able to reverse barrier dysfunctions elicited by thrombin (121). At the molecular level, S1P signaling maintains vascular integrity by cytoskeletal rearrangement and barrier enhancement through Rac activation (121). Moreover, high-density lipoproteins, acting as major plasma carriers for S1P, promote endothelial-barrier integrity via the Akt signaling pathway (122). Plasma-derived S1P also plays an essential role in maintaining vascular integrity. Indeed, mutant mice engineered to selectively lack S1P in plasma show increased vascular leak and impaired survival after administration of permeability inducing factors (123). Elevated leak was associated with interendothelial cell gaps in venules and was reversed by acute treatment with an agonist for the S1P receptor 1 (S1PR1) (123). Furthermore, recent works present S1PR1, as a key component of vascular stability (124, 125). These two studies elegantly showed that S1PR1 inhibits VEGF-induced VE-cadherin destabilization and internalization, and thereby enhances cell–cell adhesion (124, 125).

Other factors, such as FGF, can act as VE-cadherin stabilizing agents. Indeed, the inhibition of FGF signaling results in the dissociation of the VE-cadherin/p120-catenin complex, and subsequent VE-cadherin internalization, disassembly of AJ and TJ, and loss of vascular barrier integrity (126).

Thus, since they counteract VEGF-induced permeability and contribute to the maintenance of vascular barrier function, anti-permeability factors appear as potential therapeutic candidates. Another promising approach is the use of anti-VEGF/VEGF-R drugs to promote normalization of the vascular wall and its microenvironment.

### Normalization agents

From 1950 to the 2000s, the only existing non-invasive treatment for solid tumors has been chemotherapy, which is mainly based on reducing tumor cell proliferation. Because its lack of selectivity causes a large panel of side effects, new strategies, such as molecular and personalized therapies, attempt to focus on molecules overexpressed in cancers. Unfortunately, the results from clinical trials targeting such molecules in anti-cancer therapy have been quite disappointing with an overall low extension of survival, with the exception of imatinib, a tyrosine kinase inhibitor (TKI) used in chronic myelogenous leukemia treatment (127). In addition, anti-angiogenic molecules have been suggested to improve anti-cancer therapy, not only because they reduce tumor vascularization, but also thanks to their “normalization” action, which improves drug delivery.

Bevacizumab (commercialized as Avastin) was one of the first clinically available anti-angiogenic drugs. This humanized mouse antibody targeting VEGF was FDA-approved about a decade ago for combination use with standard chemotherapy in colorectal and non-small cell lung cancers. In addition, bevacizumab alone can significantly curb disease progression in patients with metastatic renal cell cancer (128). Recently published data suggest promising clinical efficacy of bevacizumab monotherapy in metastatic melanoma (129). Beside anti-VEGF, broad-spectrum multi-target TKI prolong cancer-free survival by collectively decreasing tumor vessel diameter, density, and permeability, even when administered in the absence of conventional therapies. For instance, sunitinib and sorafenib monotherapies appear particularly efficient in gastrointestinal and renal cancers (130). Nevertheless, both bevacizumab and TKI can cause serious adverse effects, such as gastrointestinal perforations.

The Notch ligand delta-like 4 (Dll4) has recently emerged as a critical regulator of tumor angiogenesis (131). Activation of the Notch pathway in neighboring endothelial cells causes inhibition of tip cell formation, an early event in sprouting angiogenesis. Mechanistically, this is believed to occur through the down-regulation of VEGF-R2/3 pro-angiogenic pathway and the up-regulation of VEGF-R1 anti-angiogenic pathway (132). Interestingly, VEGF can also operate upstream of Dll4 to potentiate its effects (133). However, the exact role of Dll4 in tumor growth and its potential in anti-cancer therapy remain unclear. Indeed, Dll4/Notch activation reduces overall tumor angiogenesis, while tumor vascular function was improved and tumor growth was heightened (134). This supports the notion that further strategy in anti-cancer therapy could be based on Dll4/Notch signaling blockade. On the other hand, Dll4-driven Notch activation might reduce both tumor-induced angiogenesis and endothelial cell responsiveness to VEGF (135), and therefore argue rather for the use of Dll4 as an effective therapeutic approach in cancers.

### Combination therapy and future strategies

Recently, based on general hallmarks of the tumor vasculature, i.e., poor blood flow, leakage, and reduced drug uptake, a new trend in anti-cancer treatment has emerged, that involves combining vascular normalization agents with traditional therapies to improve treatment response.

To re-establish an efficient tumor vascularization, assistant molecules, namely those bearing anti-angiogenic or anti-permeability properties, have been designed and tested in clinical trials, in parallel with cytotoxic drugs. In this scenario, the use of either the vasoconstrictor Angiotensin II (136) or the vasodilator Bradykinin B2 receptor agonist (137) improves tumor treatment uptake, through an increase in transcapillary pressure. Similarly, to facilitate stromal barrier crossing, the ECM-degrading enzyme collagenase (138) was shown to exert favorable changes in the transcapillary pressure gradient and thereby enhance anti-cancer drug penetration.

Alternatively, bevacizumab, the anti-VEGF drug, impinges on both microvascular density and tumor IFP, and improves drug uptake in colorectal carcinoma patients (139). Moreover, pazopanib, an inhibitor of VEGF and PDGF receptors, induces better tumor liposomal drug delivery (140). Likewise, radiotherapy combined with anti-integrin antibody (intetumumab) reduces tumor vessel density, while increasing tumor cell apoptosis and hindering metastasis (141). Interestingly, apart from its role in permeability, high levels of VEGF have been reported to promote T-reg proliferation, inhibit antigen-presenting cell maturation and as a consequence, decrease immune responses (142, 143). Therefore, anti-angiogenic drugs, especially those targeting VEGF actions, could improve cancer immunotherapy by stimulating tumor microenvironment immune responses (142). Although significant evidence has demonstrated the benefits of anti-VEGF therapies in cancer treatment, its general use is still controversial. First, significant increase in overall survival is observed only when bevacizumab is combined with standard chemotherapies. In addition, many patients exhibit resistance to anti-VEGF treatments, while timing and doses to be administered, cost and relapse effects raise some major concerns. Finally, vascular regrowth remains highly problematic. Indeed, a second wave of angiogenesis orchestrated by pro-angiogenic ligands of the FGF family could account for the short-term efficacy of VEGF-based anti-angiogenic therapies (144). Unlike bevacizumab, combination of TKI with conventional chemotherapy does not improve the outcome of anti-cancer treatment. In this scenario, use of erlotinib, a potent inhibitor of the epidermal growth factor receptor tyrosine kinase, with standard chemotherapy has failed to enhance tumor response or survival in lung carcinoma (145). Highly efficient TKI monotherapy could be combined with chemotherapy only when tumor cells become resistant to TKIs.

Alternatively, dose, schedule, and decreased toxicity may amend tumor responses. Contrary to standard chemotherapy, i.e., high doses with prolonged drug-free breaks, metronomic chemotherapy refers to chronic and equally spaced administration of low doses of cytotoxic drugs, without pauses. For example, reduced but continuous doses of cyclophosphamide suppressed tumor growth more effectively than canonical chemotherapy scheduling, even in drug-resistant tumors (146). Interestingly, metronomic chemotherapy exerts anti-tumor and anti-metastatic actions by decreasing VEGF serum concentration and increasing apoptosis of cancer cells (147). For those reasons, metronomic chemotherapy could be considered as an anti-angiogenic chemotherapy.

Importantly, the efficacy of anti-angiogenic therapy combined with cytotoxic conventional therapies (chemo- and/or radio-therapies) depends on optimal treatment scheduling. Indeed for each anti-cancer therapy, a “normalization window,” has to be determined to define period and doses necessary for tumor vessel normalization (148).

## Conflict of Interest Statement

The authors declare that the research was conducted in the absence of any commercial or financial relationships that could be construed as a potential conflict of interest.
